# Supplementary material for: Production of Proinflammatory Cytokines by CD4+ and CD8+ T Cells in Response to Mycobacterial Antigens among Children and Adults with Tuberculosis
Source: Pathogens. 2023 Nov 14;12(11):1353. doi: 10.3390/pathogens12111353 (PMC10675744; doi:10.3390/pathogens12111353)
Supplement: Supplementary file 1 [file pathogens-12-01353-s001.zip › pathogens-2650029-supplementary.pdf]

Supplemental Table S1. Median percentages of CD4+ and CD8+ T cell responsive to Mycobacterial antigens and SEB among children and adults with TB and non-TB pneumonia

	Peds Conf. TB (median; IQR)	Peds Unconf. TB (median; IQR)	Peds non-TB PNA (median; IQR)	Adult Conf. TB (median; IQR)	Adjusted p-value
CD4+ T cell: IFN-gamma					
Esat6/CFP10	0.05571 (0.19009)	0.0000 (0.00000)	0.0000 (0.00000)	0.0805 (0.11220)	<0.0001
ESXJ	0 (0)	0 (0)	0 (0)	0 (0)	0.006342
PE12/PE13	0.0000 (0.0677)	0.0000 (0.0000)	0.0000 (0.0000)	0.0619 (0.0802)	<0.0001
PE3	0.0000 (0.126525)	0.0000 (0.00000)	0.0000 (0.00000)	0.1263 (0.07080)	<0.0001
PPE15	0.0000 (0.1025)	0.0000 (0.0000)	0.0000 (0.0000)	0.1451 (0.0828)	<0.0001
PPE51	0.0000 (0.03235)	0.0000 (0.0000)	0.0000 (0.0000)	0.0557 (0.08320)	<0.0001
SEB	0.25100 (0.271325)	0.22200 (0.317000)	0.15050 (0.241400)	2.78825 (3.282775)	<0.0001
CD4+ T cell: TNF-alpha					
Esat6/CFP10	0.102135 (0.22205)	0.000000 (0.00000)	0.000000 (0.00000)	0.170800 (0.19880)	<0.0001
ESXJ	0 (0.0000)	0 (0.0000)	0 (0.0000)	0 (0.0776)	<0.0001
PE12/PE13	0.0722 (0.1117)	0.0000 (0.0000)	0.0000 (0.0000)	0.0850 (0.2080)	<0.0001
PE3	0.000 (0.1486)	0.000 (0.0000)	0.000 (0.0000)	0.209 (0.3040)	<0.0001
PPE15	0.05575 (0.185625)	0.00000 (0.000000)	0.00000 (0.000000)	0.23250 (0.280900)	<0.0001
PPE51	0.0000 (0.0736)	0.0000 (0.0000)	0.0000 (0.0000)	0.0587 (0.1376)	<0.0001
SEB	6.70725 (4.716152)	6.96418 (4.301300)	7.90370 (5.159713)	10.95350 (10.702475)	<0.0001
CD4+ T cell: IL-2					
Esat6/CFP10	0.07555 (0.24525)	0.00000 (0.00000)	0.00000 (0.00000)	0.05160 (0.11160)	<0.0001
ESXJ	0 (0)	0 (0)	0 (0)	0 (0)	0.2474
PE12/PE13	0 (0.1155)	0 (0)	0 (0)	0 (0)	0.008999
PE3	0.080300 (0.120700)	0.066825 (0.094675)	0.070500 (0.041100)	0.000000 (0.000000)	<0.0001
PPE15	0.102250 (0.061650)	0.061455 (0.097075)	0.070000 (0.084900)	0.000000 (0.052300)	<0.0001
PPE51	(0) (0.06155)	(0) (0.00000)	(0)(0.00000)	(0) 0.07120	<0.0001
SEB	0.88230 (2.111327)	0.96680 (1.266800)	0.73155 (0.637950)	9.97050 (10.990310)	<0.0001

	Peds Conf. TB (median; IQR)	Peds Unconf. TB (median; IQR)	Peds non-TB PNA (median; IQR)	Adult Conf. TB (median; IQR)	Adjusted p-value
CD4+ T cell: IFN-gamma and IL-2					
Esat6/CFP10	0.03045 (0.2110418)	0.00000 (0.0000000)	0.00000 (0. 0000000)	0.00000 (0.0621000)	0.0001419
ESXJ	0 (0)	0 (0)	0 (0)	0 (0)	0.4476
PE12/PE13	0 (0.0807)	0 (0.0000)	0 (0.0000)	0 (0.0000)	0.006617
PE3	0 (0)	0 (0)	0 (0)	0 (0)	0.3655
PPE15	0 (0)	0 (0)	0 (0)	0 (0)	0.1132
PPE51	0 (0)	0 (0)	0 (0)	0 (0)	0.07562
SEB	0.23300 (0.3954493)	0.32082 (0.4020000)	0.26325 (0.2681600)	0.88728 (1.5365700)	<0.0001
CD4+ T cell: IFN-gamma and TNF-alpha					
Esat6/CFP10	0.07615 (0.2612)	0.00000 (0.0000)	0.00000 (0.0000)	0.07316 (0.1036)	<0.0001
ESXJ	0 (0)	0 (0)	0 (0)	0 (0)	0.5434
PE12/PE13	0 (0.07575)	0 (0.00000)	0 (0.00000)	0 (0.00000)	0.001549
PE3	0.0702 (0.10200)	0.0000 (0.00000)	0.0000 (0.00000)	0.0000 (0.05311)	0.006263
PPE15	0.0686 (0.160475)	0.0000 (0.00000)	0.0000 (0.00000)	0.0000 (0.052100)	0.02991
PPE51	0 (0.11845)	0 (0.00000)	0 (0.00000)	0 (0.00000)	0.001748
SEB	0.37505 (0.480150)	0.40050 (0.671400)	0.37390 (0.395450)	1.53781 (2.324067)	<0.0001
CD4+ T cell: TNF-alpha and IL-2					
Esat6/CFP10	0.068245 (0.2758825)	0.000000 (0.0000000)	0.000000 (0.0000000)	0.000000 (0.1075000)	<0.0001
ESXJ	0 (0)	0 (0)	0 (0)	0 (0)	0.0914
PE12/PE13	0 (0.073595)	0 (0.000000)	0 (0.000000)	0 (0.000000)	0.001875
PE3	0 (0)	0 (0)	0 (0)	0 (0)	0.05899
PPE15	0 (0.01468)	0 (0.00000)	0 (0.00000)	0 (0.00000)	0.01925
PPE51	0 (0.00000)	0 (0.00000)	0 (0.00000)	0 (0.06529)	0.001141
SEB	4.403295 (2.54745)	3.435180 (3.90450)	3.526900 (2.87480)	4.857400 (7.72675)	0.02025

	Peds Conf. TB (median; IQR)	Peds Unconf. TB (median; IQR)	Peds non-TB PNA (median; IQR)	Adult Conf. TB (median; IQR)	Adjusted p-value
CD4+ T cell: IFN-gamma, TNF-alpha and IL-2					
Esat6/CFP10	0.03045 (0.2002918)	0.00000 (0.0000000)	0.00000 (0.0000000)	0.00000 (0.0593000)	0.0001358
ESXJ	0 (0)	0 (0)	0 (0)	0 (0)	0.4467
PE12/PE13	0 (0.059)	0 (0.000)	0 (0.000)	0 (0.000)	0.007623
PE3	0 (0)	0 (0)	0 (0)	0 (0)	0.3655
PPE15	0 (0)	0 (0)	0 (0)	0 (0)	0.1296
PPE51	0 (0)	0 (0)	0 (0)	0 (0)	0.07562
SEB	0.1795 (0.2863668)	0.2590 (0.3170000)	0.1980 (0.1965915)	0.6520 (1.2294370)	<0.0001
CD8+ T cell: IFN-gamma					
Esat6/CFP10	0.03515 (0.1525175)	0 (0)	0 (0)	0.06640 (0.2182000)	<0.0001
ESXJ	0 (0.065725)	0 (0)	0 (0)	0 (0)	0.04557
PE12/PE13	0 (0)	0 (0)	0 (0)	0 (0.0665)	0.001425
PE3	0.13050 (0.096325)	0.09145 (0.062050)	0.08920 (0.047295)	0.13830 (0.090800)	0.001455
PPE15	0.1378 (0.13270)	0.0783 (0.05575)	0.0876 (0.06285)	0.1369 (0.13680)	0.003341
PPE51	0 (0)	0 (0)	0 (0)	0 (0.0562)	<0.0001
SEB	4.8587 (4.32340)	3.3616 (3.26270)	2.6302 (2.12060)	6.3018 (7.35015)	<0.0001
CD8+ T cell: TNF-alpha					
Esat6/CFP10	0.10115 (0.59427)	0 (0)	0 (0)	0.11990 (0.32200)	<0.0001
ESXJ	0 (0.018475)	0 (0)	0 (0)	0 (0.052800)	0.002138
PE12/PE13	0 (0.05735)	0 (0)	0 (0)	0.0674 (0.15100)	<0.0001
PE3	0 (0.2642)	0.0536 (0.0952)	0 (0.0703)	0.1397 (0.2629)	0.0007363
PPE15	0.0520 (0.141425)	0.0512 (0.103275)	0.0570 (0.100850)	0.1468 (0.319000)	0.006588
PPE51	0 (0.02825)	0 (0)	0 (0)	0 (0)	0.1573
SEB	2.63295 (2.902925)	1.78800 (2.967400)	1.30670 (2.638350)	4.91950 (5.178275)	<0.0001

	Peds Conf. TB (median; IQR)	Peds Unconf. TB (median; IQR)	Peds non-TB PNA (median; IQR)	Adult Conf. TB (median; IQR)	Adjusted p-value
CD8+ T cell: IL-2					
Esat6/CFP10	0 (0)	0 (0)	0 (0)	0 (0)	0.6957
ESXJ	0 (0)	0 (0)	0 (0)	0 (0)	0.03083
PE12/PE13	0 (0)	0 (0)	0 (0)	0 (0)	0.7464
PE3	0 (0)	0 (0)	0 (0)	0 (0)	0.1994
PPE15	0 (0)	0 (0)	0 (0)	0 (0)	0.01367
PPE51	0 (0)	0 (0)	0 (0)	0 (0)	0.4947
SEB	0.293430 (0.834800)	0.448800 (0.635300)	0.500005 (0.714030)	0.986470 (1.576855)	0.0006457
CD8+ T cell: IFN-gamma and IL-2					
Esat6/CFP10	0 (0)	0 (0)	0 (0)	0 (0)	0.2834
ESXJ	0 (0)	0 (0)	0 (0)	0 (0)	NA
PE12/PE13	0 (0)	0 (0)	0 (0)	0 (0)	0.5524
PE3	0 (0)	0 (0)	0 (0)	0 (0)	0.03083
PPE15	0 (0)	0 (0)	0 (0)	0 (0)	0.01071
PPE51	0 (0)	0 (0)	0 (0)	0 (0)	0.5426
SEB	0 (0.063620)	0 (0.113000)	0 (0.065535)	0.21302 (0.362390)	<0.0001
CD8+ T cell: IFN-gamma and TNF-alpha					
Esat6/CFP10	0.0353 (0.15145)	0 (0)	0 (0)	0 (0.12255)	<0.0001
ESXJ	0 (0)	0 (0)	0 (0)	0 (0)	0.03616
PE12/PE13	0 (0)	0 (0)	0 (0)	0 (0)	0.8104
PE3	0 (0.0906625)	0 (0)	0 (0)	0 (0)	0.009815
PPE15	0 (0.0127)	0 (0)	0 (0)	0 (0)	0.108
PPE51	0 (0.0353)	0 (0)	0 (0)	0 (0)	0.002409
SEB	1.71110 (1.905750)	0.73030 (1.521600)	0.63943 (0.705985)	2.13662 (3.757270)	<0.0001
CD8+ T cell: TNF-alpha and IL-2					
Esat6/CFP10	0 (0)	0 (0)	0 (0)	0 (0)	0.2826
ESXJ	0 (0)	0 (0)	0 (0)	0 (0)	NA
PE12/PE13	0 (0)	0 (0)	0 (0)	0 (0)	0.5524
PE3	0 (0)	0 (0)	0 (0)	0 (0)	0.5377
PPE15	0 (0)	0 (0)	0 (0)	0 (0)	NA
PPE51	0 (0)	0 (0)	0 (0)	0 (0)	0.5426
SEB	0.08145 (0.307000)	0.10247 (0.214000)	0.08620 (0.181895)	0.31688 (0.631000)	<0.0001
CD8+ T cell: IFN-gamma, TNF-alpha and IL-2					
Esat6/CFP10	0 (0)	0 (0)	0 (0)	0 (0)	0.415
ESXJ	0 (0)	0 (0)	0 (0)	0 (0)	NA
PE12/PE13	0 (0)	0 (0)	0 (0)	0 (0)	NA
PE3	0 (0)	0 (0)	0 (0)	0 (0)	0.5377
PPE15	0 (0)	0 (0)	0 (0)	0 (0)	NA
PPE51	0 (0)	0 (0)	0 (0)	0 (0)	NA
SEB	0 (0)	0 (0.0616)	0 (0)	0.108 (0.2280)	<0.0001

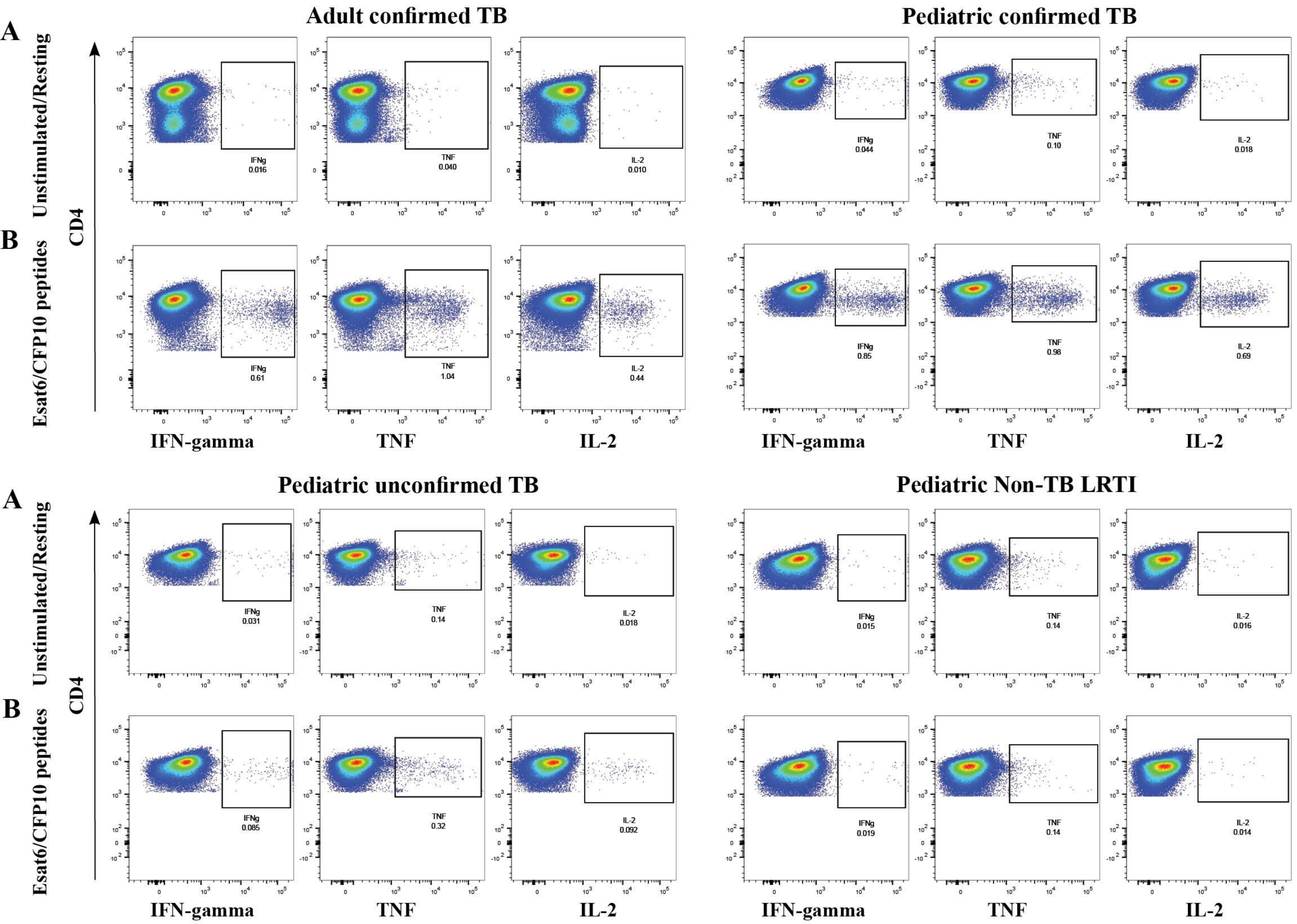

Supplemental Figure S1. Representative data demonstrating intracellular production of IFN-gamma, TNF-alpha and IL-2 by CD3+CD4+ T cells. PBMC were thawed in batches, rested overnight, and CD3+CD4+ T cell production of IFN-γ, TNF-alpha and IL-2 in unstimulated (A) and ESAT-6/CFP-10 stimulated (10 µg/ml) cells analyzed by intracellular flow cytometry following 18 hours of incubation. Shown are representative data from an adult with confirmed TB, and children with confirmed and unconfirmed TB, as well as non-TB LRTI.
